# Preclinical systematic review of ginsenoside Rg1 for cognitive impairment in Alzheimer’s disease

**DOI:** 10.18632/aging.202619

**Published:** 2021-03-03

**Authors:** Hai-Yong Liang, Pei-Pei Zhang, Xi-Le Zhang, Yan-Yan Zheng, Yan-Ran Huang, Guo-Qing Zheng, Yan Lin

**Affiliations:** 1 Department of Neurology, The Second Affiliated Hospital and Yuying Children’s Hospital of Wenzhou Medical University, Wenzhou 325000, China

**Keywords:** ginsenoside Rg1, Alzheimer's disease, learning and memory, preclinical evidence, potential mechanisms

## Abstract

Ginseng has been used for the treatment of aging and memory impairment for thousands of years. Several studies have found that ginsenoside Rg1, as one of the main active components of ginseng, could potentially improve cognitive function in several different animal models. A preclinical systematic review to evaluate the efficacy and mechanisms of ginsenoside Rg1 for ameliorating cognitive impairments in Alzheimer’s disease is reported here. We searched six databases from their inceptions to January 2019. Thirty-two studies were selected, which included a total of 1,643 animals. According to various cognitive behavioral tests, the results of the meta-analyses showed that ginsenoside Rg1 significantly improved cognitive behavioral impairments in most Alzheimer’s disease models (*P* < 0.05), but there were no significant effects in animals with neuronal degeneration induced by chronic stress or in SAMP8 transgenic mice. The potential mechanisms included antioxidant and anti-inflammatory effects, amelioration of Alzheimer’s disease-related pathology, synapse protection, and up-regulation of nerve cells via multiple signaling pathways.

## INTRODUCTION

Cognitive function is important to human life, but it is vulnerable across a person’s lifetime [1]. Many diseases, such as Alzheimer’s disease (AD), vascular dementia, and depression [2–4], can lead to cognitive impairment and finally destroy a person’s ability to function in daily life [5]. Aging is the most important factor in all causes of dementia [6]. With the increasingly aging worldwide population [7], the prevalence of dementia is set to approximately double every 20 years, and the number of people living with dementia is expected to reach 66 million in 2030 and 115 million in 2050 [8]. The estimated annual worldwide cost of dementia is $604 billion, approximately 1% of the world’s gross domestic product, which represents a huge financial burden to human society [9]. Moreover, the caregivers of dementia patients can also suffer from physical and psychological problems [10]. Cholinesterase inhibitors and memantine are used to treat dementia [11]; however, it has been found that the benefit of these drugs might be minor overall [12, 13]. Thus, it is high time to seek a novel approach for prevention and/or treatment of cognitive impairment, especially that caused by AD.

**Figure 1:**
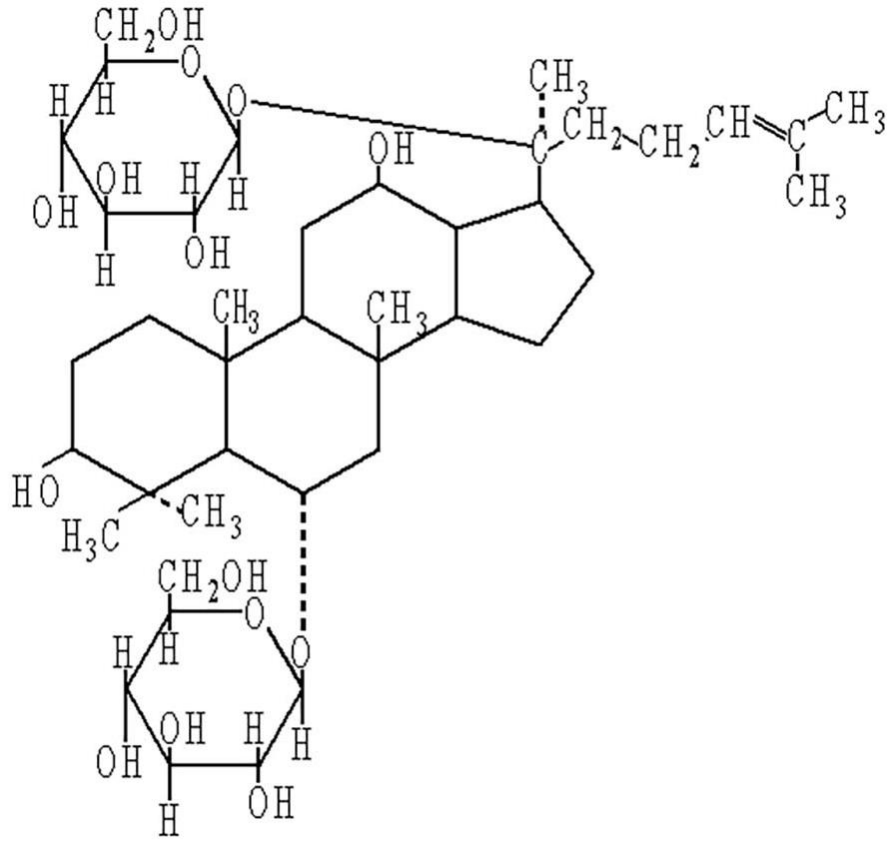
**Chemical structure of ginsenoside Rg1.**

For thousands of years, traditional Chinese medicine has taken a holistic approach to illness [14]. Ginseng, the root and rhizome of *Panax ginseng* C.A. Mey, is known as an adaptogenic herb [15]. It has traditionally been used in East Asian countries for more than 2,000 years for the treatment of aging and memory impairment [16]. More recently, use of ginseng has been increasing around the world [17]. Recent studies have also confirmed the effect of ginseng in neurological and neurodegenerative disorders [18]. Ginsenosides are important active components of ginseng and are responsible for its major effects [19]. Ginsenoside Rg1 (G-Rg1) is the most abundant and active ginsenoside, and it has a structure similar to that of steroid hormones [20] (see Figure 1). It is believed that G-Rg1 could cross the blood–brain barrier and exert potential neuroprotective effects [21, 22]. Numerous studies have suggested that G-Rg1 could improve cognitive function in different animal models, and most of these have been AD models. However, the underlying mechanisms of G-Rg1 on AD are poorly understood.

A systematic review of animal-based studies can provide transitional value to the treatment of human diseases, as well as highlighting potential limitations and hidden innovative strategies in animal experiments [23, 24]. A previous review focused on different ginsenosides and their effects on AD, but this review included only 12 articles, and only five of these were G-Rg1 studies [25]. Therefore, it is important that a G-Rg1-specific study should be conducted. In the present study, we considered 32 studies examining the effects of G-Rg1 in AD.

## RESULTS

### Study inclusion

**Figure 2:**
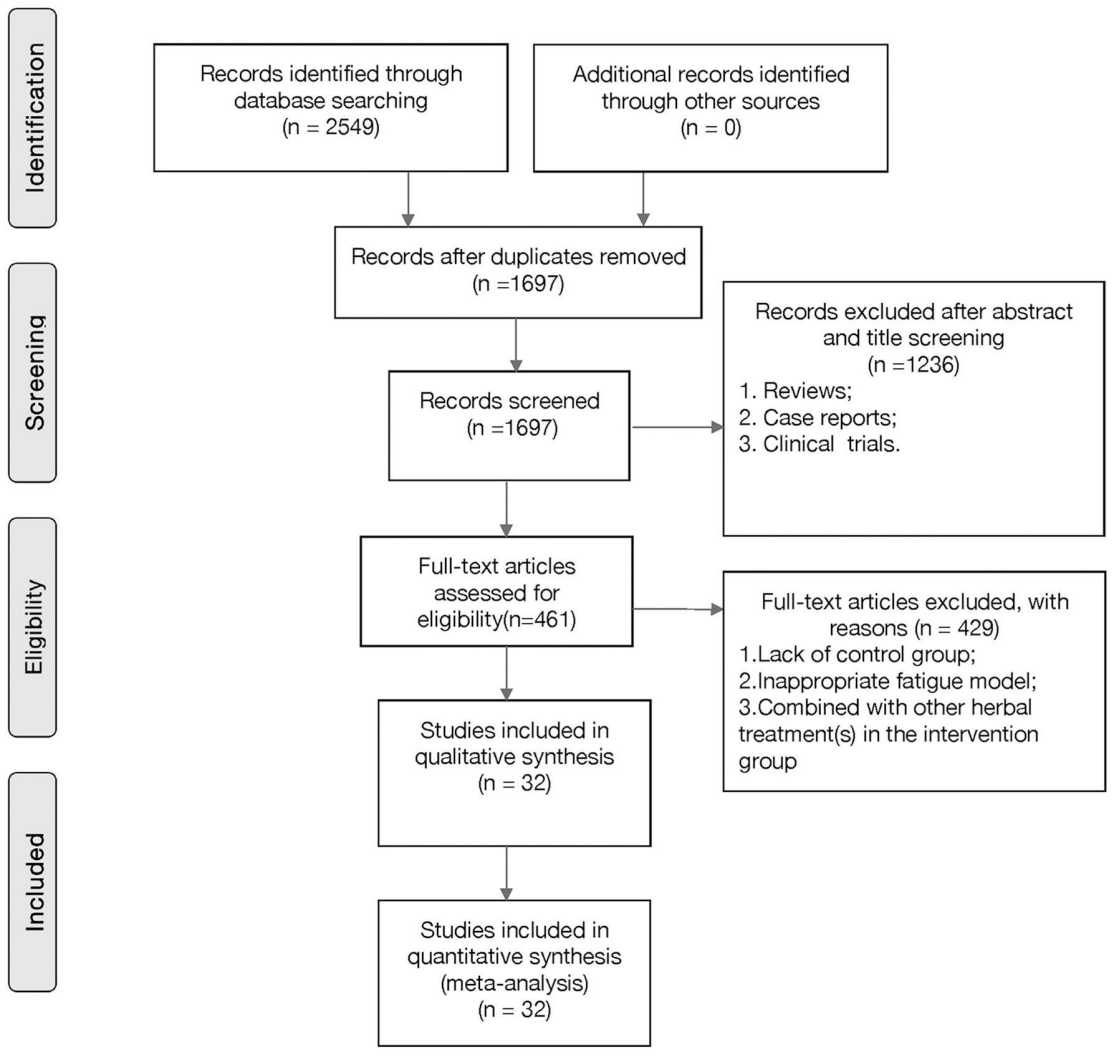
**Flow diagram of the study-search process.**

A total of 2,549 hits were found in an electronic database search, of which 852 duplicates were removed. We then screened titles and abstracts, and 1,236 further studies were excluded because they were clinical trials, case reports, or review articles. Through full-text evaluation of the remaining 461 studies, 429 were excluded for at least one of the following reasons: (1) unavailability of data; (2) not predetermined outcome index; (3) no *in vivo* model; (4) no control group; or (5) the intervention group did not receive G-Rg1 as a monotherapy. Eventually, 32 studies were selected (Figure 2).

### Characteristics of included studies

All included studies were published between 2001 and 2018. Of these, 16 were published in Chinese and 16 were published in English. These studies used ten different animal species, of which nine studies used Sprague Dawley rats, five used Wistar rats, three used Kunming mice, three used C57BL/6J mice, three used APP/PS1 mice, two used nestin-GFP mice, two used ICR mice, two used SAMP8 mice, one used APP/PS1/tau mice, and one used mAPP mice. As for the animal model, these studies used AD animal models including transgenic mice (*n* = 8), aged mice (*n* = 1), ovariectomy plus intracranial injections of d-gal (*n* = 1), hippocampus injury (*n* = 3), chronic stress (*n* = 2), ovariectomy (*n* = 1), and injection of okamoto acid (*n* = 1), quinolinic acid (*n* = 1), the amyloid β (Aβ) 1-42 (*n* = 3) and Aβ25-35 (*n* = 2), dexamethasone (*n* = 1), d-gal (*n* = 6), and scopolamine (*n* = 2). Twenty-six studies used Morris water maze (MWM) as an index of cognitive function, of which 25 reported escape latency (EL) to represent the spatial test and 17 reported the number of platform crossings (NOPCs) to represent the probe test. Four studies used a Y maze, three used a step-down test, one used a dark-avoidance test, one used a fear conditioning test, one used a water maze, one used a novel-object-recognition test, and one used a radial-arm water maze. The characteristics of the included studies are shown in Supplementary Tables 1, 2.

### Study quality

The study quality scores ranged from 1/10 to 7/10, with a mean score of 4.03/10. Twenty-nine studies were published in peer-reviewed publications, while three were master's or doctoral theses. Fifteen of the 32 studies reported controlling the temperature. Twenty-five studies reported random grouping of experimental animals. Twelve studies used an anesthetic, of which four were considered to have a slight influence on cognitive function. Compliance with animal welfare regulations was reported in eight studies. Sixteen of the studies had a statement of potential conflicts of interest. The application of blind methods in the induction of the model or the assessment of the outcome was not reported in any studies, nor were calculations for sample sizes reported (see Table 1).

**Table 1:** The methodological quality of included studies.

**Study**	**A**	**B**	**C**	**D**	**E**	**F**	**G**	**H**	**I**	**J**	**Total**
Chen et al. 2005	√	×	√	×	×	√	√	×	×	×	4
Chen et al. 2011	√	×	√	×	×	×	√	×	×	×	3
Chen et al. 2017	√	×	√	×	×	√	×	×	√	√	5
Fang et al. 2012	√	×	×	×	×	√	×	×	√	√	4
Hu et al. 2004	√	×	√	×	×	√	×	×	×	×	3
Li et al. 2007	√	×	√	×	×	√	√	×	×	×	4
Li et al. 2014	√	×	√	×	×	√	×	×	×	×	3
Li et al. 2015	√	√	√	×	×	√	×	×	×	√	5
Li et al. 2016a	√	√	×	×	×	√	×	×	×	√	4
Li et al. 2016b	√	√	×	×	×	√	×	×	×	√	4
Liu et al. 2015	×	×	√	×	×	√	×	×	×	×	2
Nie et al. 2017	√	×	×	×	×	√	√	×	×	√	4
Peng et al. 2011	√	√	√	×	×	√	×	×	×	×	4
Quan et al. 2013	√	√	√	×	×	√	×	×	×	√	5
Shi et al. 2008	×	√	√	×	×	√	√	×	×	×	4
Shi et al. 2012	√	√	×	×	×	?	√	×	×	√	4
Shi et al. 2018	√	√	√	×	×	√	√	×	√	√	6
Song et al. 2013	√	√	√	×	×	√	×	×	√	√	5
Wang et al. 2001	√	×	×	×	×	×	×	×	×	×	1
Wang et al. 2010	√	√	×	×	×	√	×	×	√	√	4
Wang et al. 2014b	√	×	√	×	×	√	×	×	√	√	5
Wu et al. 2007	√	×	√	×	×	√	×	×	×	×	3
Wu et al. 2011	√	×	√	×	×	√	×	×	×	×	3
Xiang et al. 2017	√	√	√	×	×	√	×	×	×	×	4
Yang et al. 2013	×	√	√	×	×	√	√	×	×	×	4
Ye et al. 2017	√	×	√	×	×	?	×	×	×	×	2
Yuan et al. 2016	√	×	√	×	×	√	×	×	×	×	3
Zhang et al. 2012	√	√	√	×	×	√	√	×	×	√	6
Zhang et al. 2017a	√	√	√	×	×	√	×	×	√	√	7
Zhang et al. 2017b	√	×	√	×	×	√	×	×	×	√	4
Zhou et al. 2011	√	√	√	×	×	√	√	×	×	×	5
Zhu et al. 2014	√	×	√	×	×	√	×	×	√	√	5

Note: Studies fulfilling the criteria of: A: peer reviewed publication; B: control of temperature; C: random allocation to treatment or control; D: blinded induction of model; E: blinded assessment of outcome; F: use of anesthetic without significant intrinsic neuroprotective activity; G: animal model (aged or female involved); H: sample size calculation; I: compliance with animal welfare regulations; J: statement of potential conflict of interests.

### Effectiveness

**Figure 3:**
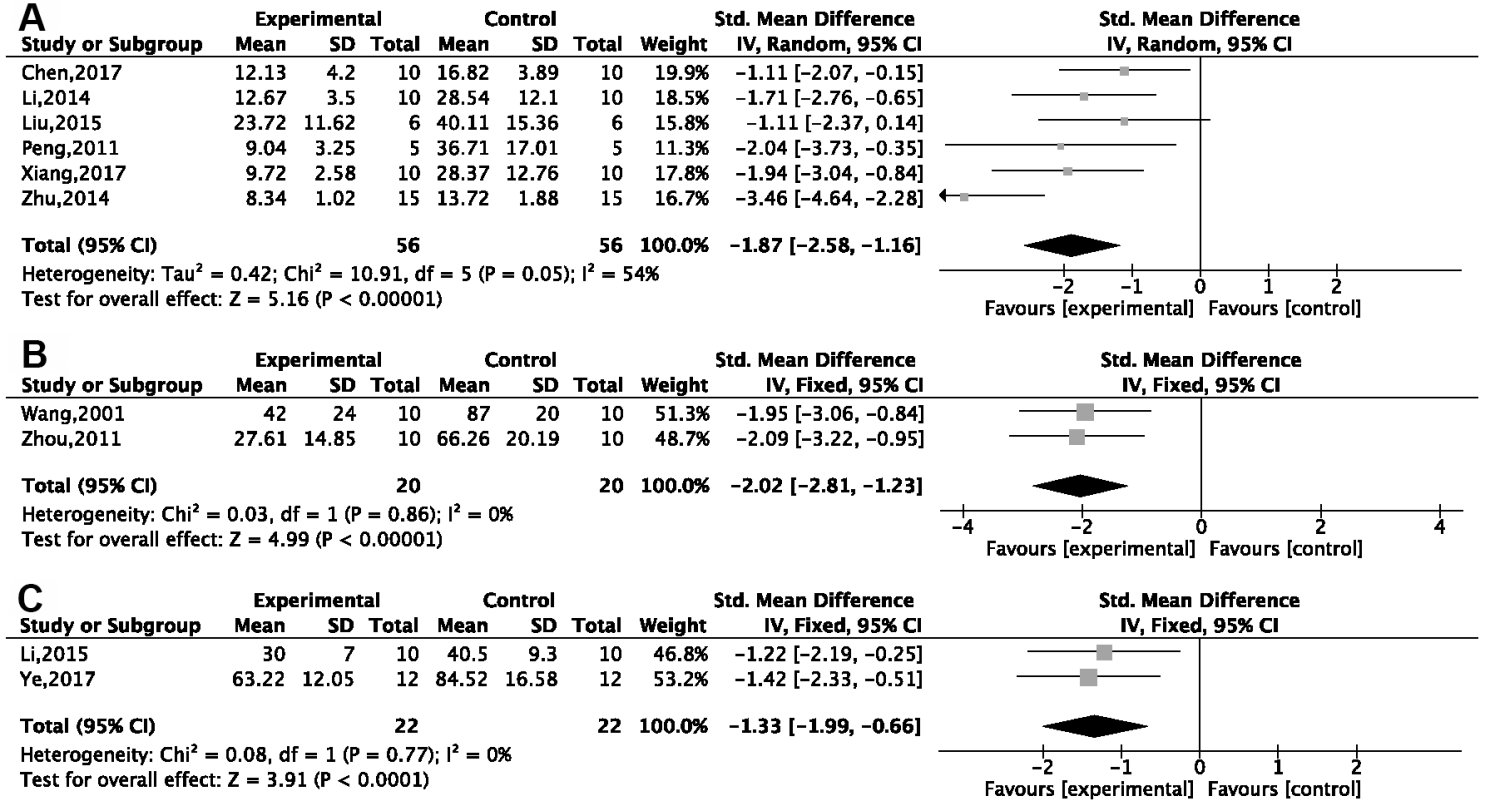
**Forest plots of escape latency for the Morris water maze.** This was seen to decrease in (**A**) the d-gal injection model; (**B**) the Aβ25-35 injection model; and (**C**) the Aβ1-42 injection model in the ginsenoside Rg1 group compared with a control group.

The MWM was used in 26 studies, of which 23 reported the EL in the spatial test. Due to notable heterogeneity, we conducted a subgroup analysis based on the different animal modeling methods. Compared with normal saline or no treatment, G-Rg1 was found to decrease EL in a statistically significant way in a meta-analysis of six studies using a d-gal injection model [26–31] {*P* < 0.00001; standardized mean difference (SMD) = −1.87, 95% confidence interval (CI) [−2.58, −1.16]; heterogeneity: *χ*^2^ = 10.91, df = 5 (*P* = 0.05); I^2^ = 54%, Figure 3A}; two studies [32, 33] using an Aβ25-35 injection model {*P* < 0.00001; SMD = −2.02, 95%CI [−2.81, −1.23]; heterogeneity: *χ*^2^ = 0.03, df = 1 (*P* = 0.86); I^2^ = 0%, Figure 3B}; and three studies [34–36] using an Aβ1-42 injection model {*P* < 0.00001; SMD = −1.70, 95%CI [−2.31, −1.09]; heterogeneity: *χ*^2^ = 7.98, df = 2 (*P* = 0.02); I^2^ = 75%}. Due high heterogeneity, we conducted a sensitivity analysis. After omitting one study, the results of two studies [35, 36] showed good homogeneity {*P* < 0.00001; SMD = −1.33, 95%CI [−1.99, −0.66]; heterogeneity: *χ*^2^ = 0.08, df = 1 (*P* = 0.77); I^2^ = 0%, Figure 3C}. Other methods of modeling, including hippocampal resection (*n* = 2), hippocampus electrical injury (*n* = 1), ovariectomy plus injection of d-gal (*n* = 1), using aged mice (*n* = 1), APP/PS1/tau transgenic mice (*n* = 1), and injection of okamoto acid (*n* = 1) and scopolamine (*n* = 1), all showed G-Rg1 could significantly decrease EL (*P* < 0.05). The effect that G-Rg1 has on EL in SAMP8 transgenic mice and chronic stress mice is controversial; that is, two studies [37, 38] showed significant differences comparing the treatment group with the control, while two other studies [39, 40] did not.

**Figure 4:**
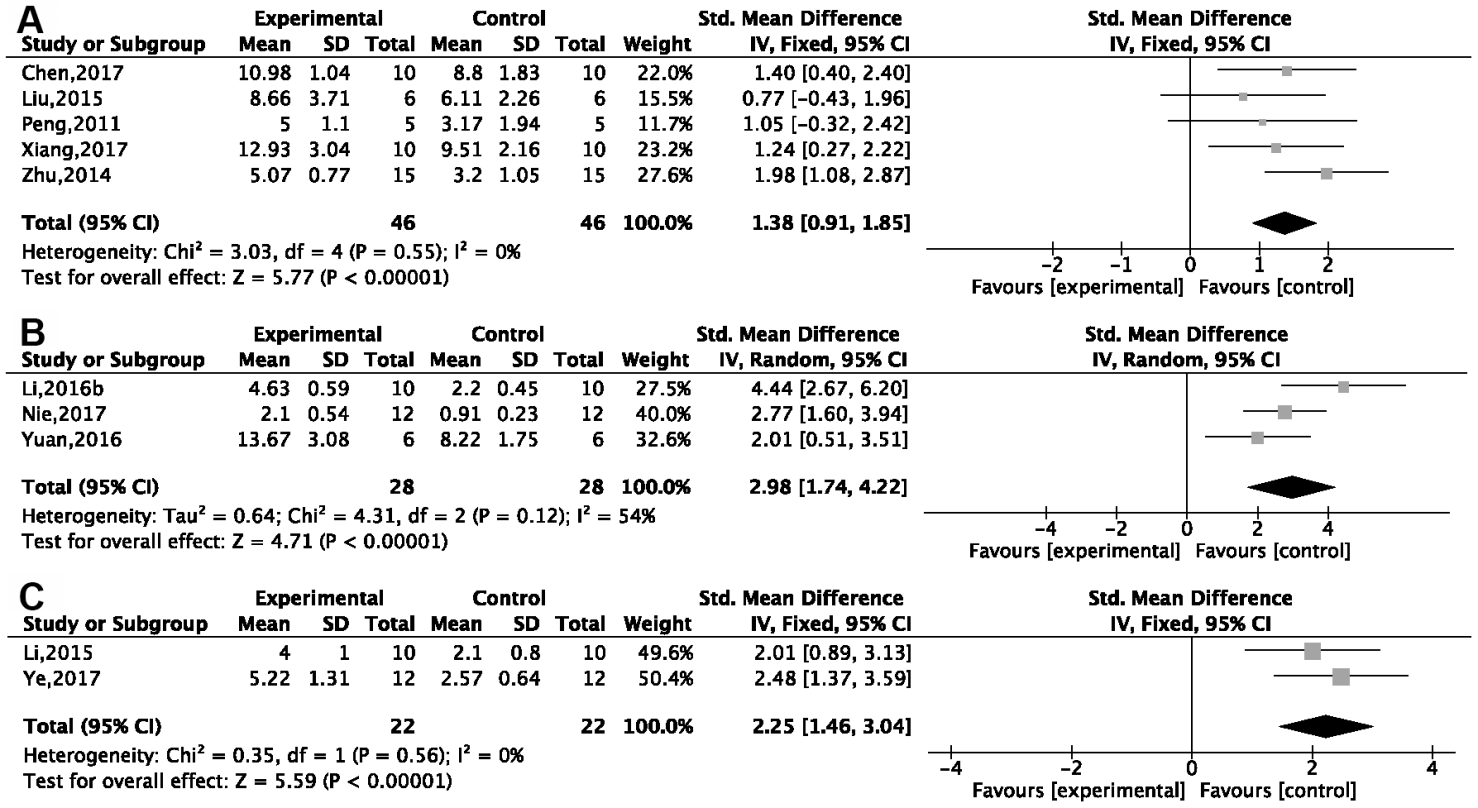
**Forest plots of the number of platform crossings in the Morris water maze.** Improvements were seen in (**A**) the d-gal injection model; (**B**) APP/PS1 transgenic mice; (**C**) the Aβ1-42 injection model in the ginsenoside Rg1 group compared with a control group.

Twenty-one studies reported the NOPC in the probe test. Subgroup analysis based on different methods of animal modeling showed significant improvement of platform crossings in the G-Rg1 group compared with the control group, of which five studies used a d-gal injection model [26, 27, 29–31] {*P* < 0.00001; SMD = 1.38, 95%CI [0.91, 1.95]; heterogeneity: *χ*^2^ = 3.03, df = 4 (*P* = 0.55); I^2^ = 0%, Figure 4A}; two studies [41–43] used APP/PS1 transgenic mice {*P* < 0.00001; SMD = 2.98, 95%CI [1.74, 4.22]; heterogeneity: *χ*^2^ = 4.31, df = 2 (*P* = 0.12); I^2^ = 54%, Figure 4B}; and two studies [35, 36] used Aβ1-42 injection model {*P* < 0.00001; SMD = 2.25, 95%CI [1.46, 3.04]; heterogeneity: *χ*^2^ = 0.35, df = 1 (*P* = 0.56); I^2^ = 0%, Figure 4C}. Other methods of modeling, such as using SAMP8 transgenic mice (*n* = 2), Aβ25-35 injection (*n* = 1), scopolamine injection (*n* = 2), chronic stress (*n* = 1), okamoto acid injection (*n* = 1), and ovariectomy plus d-gal injection (*n* = 1), showed G-Rg1 could increase the NOPC (*P* < 0.05).

In four studies, Y-maze tests were conducted. One study [44] showed that G-Rg1 could improve the correct response rates in learning and memory tests (*P* < 0.05). By comparison with the control group, one study [45] found that G-Rg1 increased spontaneous alternation (*P* < 0.05), one study [39] found that it decreased the number of trials reaching the criterion (*P* < 0.05), and one study [46] found that it decreased the error times (*P* < 0.05).

Due to high statistical heterogeneity, we merely conducted a systematic review. Three studies [46–48] conducted step-down tests according to reported latency and/or the number of errors, and these showed positive results (*P* < 0.05) compared with a control. A radial-arm water-maze test, fear-conditioning experiment, dark-avoidance test, and novel-object-recognition test were each carried out individually in four studies [32, 49–51], and all showed that G-Rg1 could significantly improve cognitive function compared with a control (*P* < 0.05).

**Figure 5:**
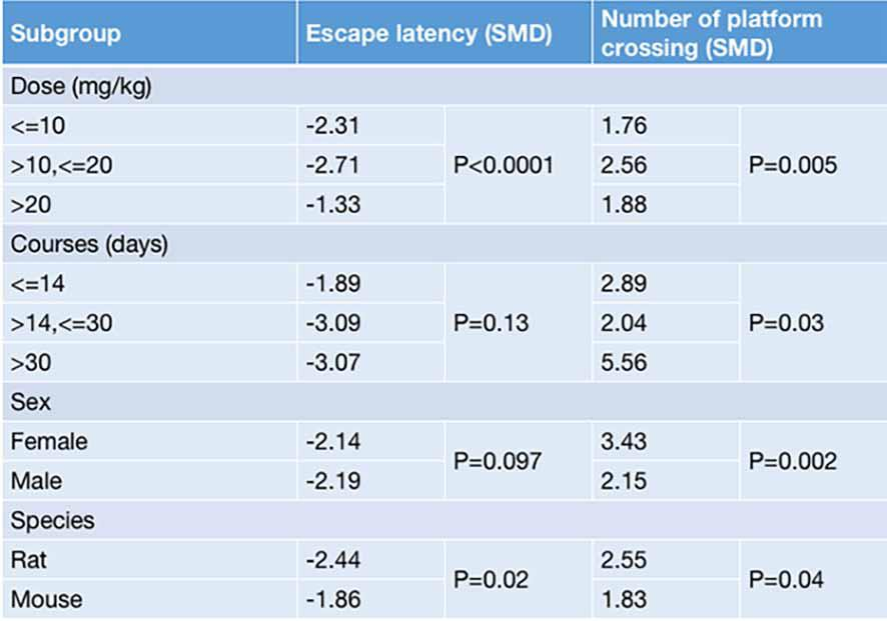
**Results of the stratified meta-analysis regarding escape latency and the number of platform crossings in the Morris water maze.**

Stratified analyses of the EL and the NOPC were conducted based on the variables involved in the dosage and treatment course of G-Rg1, the animal species, and the animal sex, aiming to explore potential methodological differences that may have affected the treatment outcomes. For both the EL and the NOPC, a dosage greater than 10 mg/kg and less than 20 mg/kg was associated with more positive outcomes compared with a dosage less than 10 mg/kg or more than 20 mg/kg (EL: SMD_≤10 mg/kg_ = −2.31, SMD_>10,≤20 mg/kg_ = _−_2.71, SMD_>20 mg/kg_ = −1.33, *P* < 0.0001; NOPC: SMD_≤10 mg/kg_ = 1.76, SMD_>10,≤20 mg/kg_ = 2.56, SMD_>20 mg/kg_ = 1.88, *P* = 0.05. Figure 5). There was a significant difference in the effect of G-Rg1 on decreasing the NOPC for different treatment courses, but no significant difference on improving the EL (EL: SMD_≤14 days_ = −1.89, SMD_>14,≤30 days_ = _−_3.09, SMD_>30 days_ = −3.07, *P* =0.13; NOPC: SMD_≤14 days_ = 2.89, SMD_>14,≤30 days_ = 2.04, SMD_>30 days_ = 5.56, *P* = 0.03. Figure 5). The effect of G-Rg1 was greater in rat species than mice species for both the EL and the NOPC (EL: SMD_rat_ = −2.44, SMD_mice_ = −1.86, *P* = 0.02; NOPC: SMD_rat_ = 2.55, SMD_mice_ = 1.83, *P* = 0.04, Figure 5). In the subgroup analysis of animal sex, the effect of G-Rg1 on the NOPC was significantly larger in female mice (SMD_female_ = 3.43, SMD_male_ = 2.15, *P* = 0.002, Figure 5), whereas there was no significant difference in the EL (SMD_female_ = −2.14, SMD_male_ = −2.19, *P* = 0.097, Figure 5).

### Mechanisms

### *Anti-oxidation effects*

**Figure 6:**
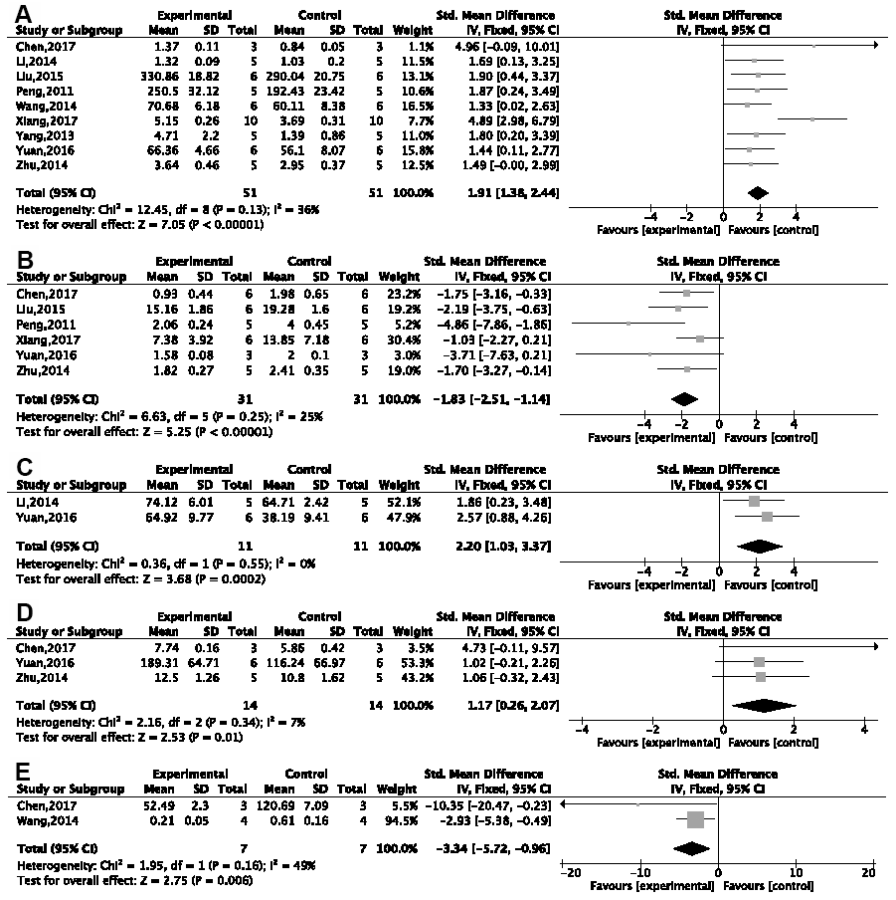
**Forest plots of the effect of ginsenoside Rg1 for anti-oxidation.** Ginsenoside Rg1 (**A**) improved the activity of SOD; (**B**) decreased the level of MDA; (**C**) improved the activity of GSH; (**D**) improved the activity of GSH-PX; (**E**) decreased the level of ROS compared with a control group.

A remarkable effect of G-Rg1 in increasing the activity of superoxide dismutase (SOD) was seen in a meta-analysis of nine studies [26–31, 39, 41, 45] {*P* < 0.00001; SMD = 1.91, 95%CI [1.38, 2.44]; heterogeneity: *χ*2 = 12.45, df = 8 (*P* = 0.13); I^2^ = 36%, Figure 6A}; six studies [26, 27, 29–31, 41] in decreasing malondialdehyde (MDA) {*P* < 0.00001; SMD = −1.83, 95%CI [−2.51, −1.14]; heterogeneity: *χ*2 = 6.63, df = 5 (*P* = 0.25); I^2^ = 25%, Figure 6B}; and three studies [26, 28, 41] in increasing glutathione (GSH) {*P* < 0.0001; SMD = 2.51, 95%CI [1.36, 3.65]; heterogeneity: *χ*^2^ = 7.19, df = 2 (*P* = 0.03); I^2^ = 72%}; and a sensitivity analysis of two studies [28, 41] showed good heterogeneity {*P* = 0.0002; SMD = 2.20, 95%CI [1.03, 3.37]; heterogeneity: *χ*^2^ = 0.36, df = 1 (*P* = 0.55); I^2^ = 0%, Figure 6C}. By comparison with the control group, meta-analysis of three studies [26, 31, 41] showed that G-Rg1 increases the level of glutathione peroxidase {*P* = 0.01; SMD = 1.17, 95%CI [0.26, 2.07]; heterogeneity: *χ*^2^ = 2.16, df = 2 (*P* = 0.34); I^2^ = 7%, Figure 6D} and two studies [31, 39] showed a decrease in reactive oxygen species (ROS) {*P* = 0.006; SMD = −3.34, 95%CI [−5.72, −0.96]; heterogeneity: *χ*^2^ = 1.95, df = 1 (*P* = 0.16); I^2^ = 49%, Figure 6E}.

### Anti-inflammatory effects

**Figure 7:**
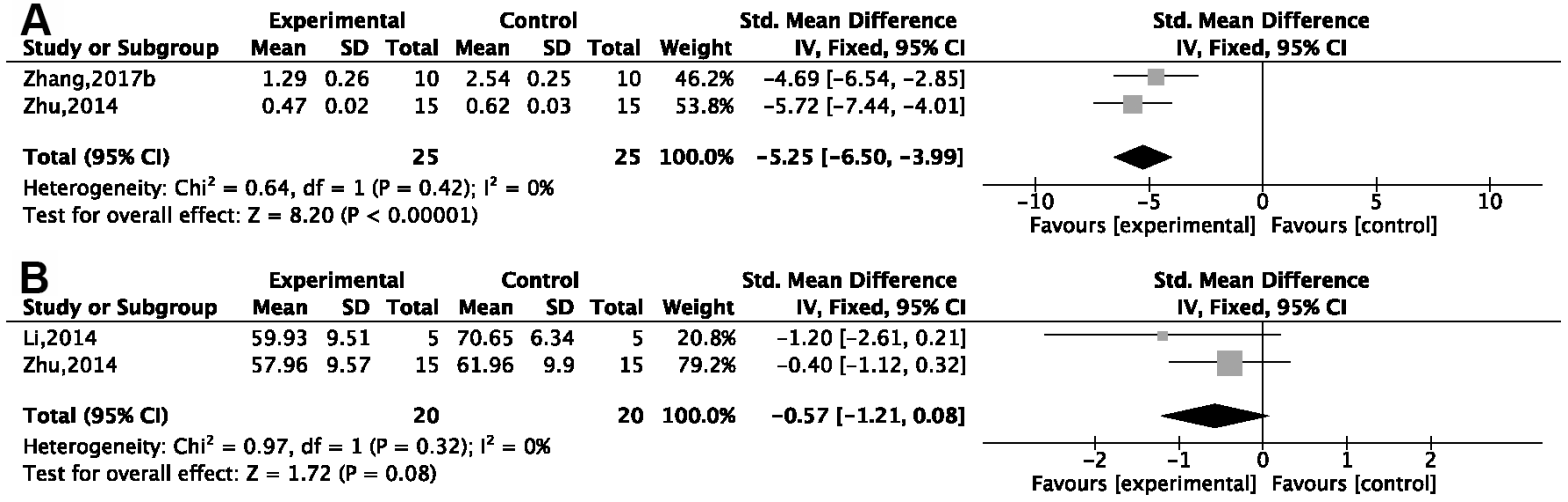
Forest plots showing that ginsenoside Rg1 decreased the content of (**A**) IL-1β and (**B**) IL-6 compared with a control.

Two studies [26, 30] showed a remarkable effect of G-Rg1 in decreasing tumor necrosis factor α (TNF-α; *P* < 0.05). Compared with the control group, one study [28] showed a decrease in interleukin (IL)-1 in the G-Rg1 group (*P* < 0.05); three studies [26, 30, 51] showed a decrease in IL-1β {*P* < 0.00001; SMD = −3.37, 95%CI [−4.19, −2.54]; heterogeneity: *χ*^2^ = 15.19, df = 2 (*P* = 0.0005); I^2^ = 87%}; and sensitivity analysis of two studies [26, 51] showed good homogeneity {*P* < 0.00001; SMD = −5.25, 95%CI [−6.50, −3.99]; heterogeneity: *χ*^2^ = 0.64, df = 1 (*P* = 0.42); I^2^ = 0%, Figure 7A}. In three studies, IL-6 was seen to decrease [26, 28, 30] {*P* < 0.00001; SMD = −0.94, 95%CI [−1.56, −0.31]; heterogeneity: *χ*^2^ = 23.99, df = 2 (*P* < 0.00001); I^2^ = 92%}. Due to notable heterogeneity, sensitivity analyses were performed, and the results showed more homogeneity [26, 28] {*P* < 0.0001; SMD = −0.57, 95%CI [−1.21, −0.88]; heterogeneity: *χ*^2^ = 0.97, df = 1 (*P* = 0.55); I^2^ = 0%, Figure 7B}. IL-18 was seen to decrease in the G-Rg1 group in one study [51] when compared with a control group.

### Up-regulation of nerve cells

Nestin is one of the widely used markers for neural stem cells (NSCs). Four included studies reported that G-Rg1 could increase the expression of nestin, of which two [29, 30] used real-time qRT-PCR and two [25, 29] used 4′,6-diamidino-2-phenylindole nuclear staining.

Senescence-associated beta-galactosidase (SA-β-gal) is one of the most commonly used biomarkers for determining the age of cells. Three studies [26, 28, 45] reported that G-Rg1 decreased the intensity of SA-β-gal stain in the brain or CA3 area (*P* < 0.05).

One study [25] used immunofluorescence of 5-bromo-2′-deoxyuridine to demonstrate that G-Rg1 could increase neurogenesis by increasing the number of new cells (*P* < 0.05). Three studies using hematoxylin–eosin staining found that G-Rg1 could alleviate neuronal damage such as nuclear condensation and acidophilic degeneration [39, 51, 52]. One study showed that G-Rg1 inhibited cell apoptosis according to TUNEL or nuclear concentrated cell numbers (*P* < 0.05) [36, 39].

### Synapse protection

**Figure 8:**
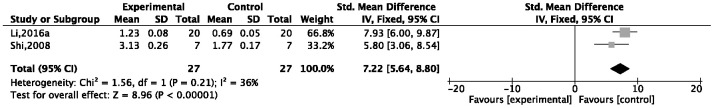
**Forest plot of the expression of BDNF for the ginsenosdie-Rg1 group versus a control group.**

Meta-analysis of two studies [40, 50] showed a remarkable effect of G-Rg1 on increasing brain-derived neurotrophic factor (BDNF) {*P* < 0.00001; SMD = 7.05, 95%CI [5.64, 8.80]; heterogeneity: *χ*^2^ = 1.56, df = 1 (*P* = 0.21); I^2^ = 36%, Figure 8}. One study [42] reported that G-Rg1 treatment could increase the expression of multiple synaptic proteins such as synaptosomal-associated protein 25 (SNP25), synapsin 2 (SYN2), and complexin 2. The results indicated that G-Rg1 may ameliorate synaptic plasticity.

Three studies showed a significant effect of G-Rg1 on decreasing the activity of acetylcholinesterase (AChE) according to absolute AChE activity [32, 44] or relative AChE activity (% of controls) [48] (*P* < 0.05). Moreover, one study [48] showed G-Rg1 could increase the content of acetylcholine (ACh) (*P* < 0.05), one study [32] showed an increase in the activity of choline acetyltransferase, and one study [48] showed an increase in the content of 5-hydroxytryptamine (*P* < 0.05) compared with the control group.

### Amelioration of AD-related pathology

Using an ELISA, one study showed decreases in Aβ1-40 in brain slices [49] and another study showed decreases in the hippocampus [46] compared with controls. One study [48] showed Aβ1-42 was decreased in brain slices, and three studies [50, 53, 54] showed decreases in the hippocampus after G-Rg1 treatment (all *P* < 0.05). In addition, one study [53] reported that G-Rg1 had a significant effect on decreasing Aβ in the hippocampus (*P* < 0.05) but not in the cortex. Two studies [35, 36] showed Aβ was decreased in the G-Rg1 group compared with the control group, according to the use of an optical microscope.

Two studies [50, 55] reported that G-Rg1 could decrease tau and another showed [50] that it could decrease APP. A study [53] showed an increase in soluble APPα (all *P* < 0.05), compared with the control group. According to one study [54], G-Rg1 could increase disintegrin and metallopeptidase domain 10 (ADAM10) expression and decrease β-secretase β-site APP-cleaving enzyme 1 (BACE1) expression, which are related to α-secretase and β-secretase. In addition, Fang et al. demonstrated G-Rg1 treatment could decrease γ-secretase activity from both *in vitro* and *in vivo* results [49].

## DISCUSSION

### Summary of evidence

This is the first preclinical systematic review that focused on evaluating the efficacy and potential mechanisms of G-Rg1 for AD. Thirty-two studies with 1,643 animals were identified. The evidence showed that G-Rg1 could improve learning and memory function, and enhance animals’ performances in MWM, Y-maze, dark-avoidance, novel-object-recognition, radial-arm water-maze, and fear-conditioning tests. The mechanisms were related to anti-oxidation, anti-inflammatory activities, amelioration of AD-related pathology, synapse protection, and up-regulation of nerve cells.

### Limitations

First, only databases in English and Chinese were searched. Korea is one of the main ginseng distributers in the world [56]; therefore, some relevant publications may have been missed.

Second, the probability of a study with positive results being published is about three times that of studies with neutral or negative results [57]. The factors contributing to publication bias are various and include researchers and editors preferring results with meaningful *P* values to those with inconclusive results [58]. In addition, some studies displayed the original data in the form of graphs, and data extracted using “digital ruler” software may be subject to slight errors. Some studies did not even show the original data. The efficacy of G-Rg1 might be overestimated because of this lack of related data.

Third, animal studies with a less rigorous design may exaggerate the real effects. The quality of the included studies was moderate. Study quality is a multidimensional concept that is related to several different factors, including a trial’s design, conduction, analyses, clinical applicability, and reporting [59]. The assessment of the risk of bias could help to avoid over- or underestimating the parameters of interest, and this is vital when interpreting study results [60]. However, using different scales for quality appraisal can lead to inconsistent results if the scales are notably different in their complexity and dimensions [61]. Furthermore, the extent to which the quality of reports reflects the quality of randomized controlled trials (RCTs) is still a matter of debate [62].

Some methodological flaws still exist. All the studies considered here failed to report the calculations for sample sizes, and having a sufficient sample size is vital for identifying the effects of a drug or therapy [63]; an insufficient sample size will result in inaccurate estimation of the treatment effect. The use of blind methods in the process of the research is not mentioned in any of the studies, and these can play an important role in the measurement and assessment of outcomes. The lack of blind methods could result in bias in performance and detection [64]. In addition, two studies used male/female models. It has been reported that male models perform well in working-memory tasks, while female models are good for visual-memory tasks and social cognition [65]. Failing to consider the sex of the animals may introduce a new uncontrolled variable that could affect outcomes. Moreover, two studies used pentobarbital sodium as an anesthetic. This anesthetic can result in some damage to cognitive function [66] and could therefore cause underestimation of the effect of G-Rg1.

Fourth, two studies did not report the source of G-Rg1, and many other studies did not report quality control or chemical analysis of the G-Rg1. By tracing the sources of G-Rg1 used in the included studies, we found that its purity reported by the suppliers ranged from >95% to>99%. A comprehensive review conducted by the European Medicines Agency in 2013 [67] found that the concentrations of isolated compounds applied in animal models are significantly higher than expected, indicating the presence of other active compounds. This may be related to the methods used for the purification of ginsenosides, such as chromatographic column separation, which can lead to other ginsenosides being present in the final sample. According to a report, the water content was found to be 0.485% for G-Rg1, with a net mass balance of 99.515% [68]. Thus, high concentrations of G-Rg1 should be obtained from the marketing authorization holder.

Finally, none of the included studies reported the number of animals that died or were removed from the study for other reasons. This information is essential for accurately assessing the usage of G-Rg1.

### Implications for research

Approximately 70% of the prevalence of dementia can be ascribed to AD [69], and this correlates with the trend of an aging population [70]. The pathogenesis of AD is not clear, and the methods for modeling the disease are diverse. The included studies used models involving intracranial injections of d-gal, Aβ1-45, Aβ25-35, dexamethasone, okamoto acid, scopolamine, and quinolinic acid, ovariectomy plus intracranial injections of d-gal, aged mice, and transgenic mice, such as APP/PS1 mice, APP/PS1/tau mice, and SAMP8 mice.

These models have both advantages and disadvantages in mimicking AD. Intracranial injections have a low cost and studies are quicker to conduct than when using transgenic animals. Moreover, injection of substances such as Aβ can localize the intended effect and exclude other confounding effects. However, intracranial injection results in an acute model, but AD is a gradually developing disease in humans [71, 72]. Transgenic models integrate genes that encode proteins associated with part of the pathology of AD. Such models could help in the understanding of regional vulnerability and pathogenesis of AD because specificity of brain areas and cells is achieved by introducing the target gene under the control of promoters and regulatory elements [73]. Since cases of AD are sporadic and have unclear etiology, the mutations that are carried most frequently by transgenic animals only account for <5% of all AD cases [74]. Due to the complex mechanisms of human AD, it is of great importance to accept that all currently available models fail to replicate the full-scale features of AD [75]. However, some essential questions about the pathophysiology of AD have been resolved using the animal models available today [76].

After mastering enough knowledge of animal models and their intrinsic limitations, it is possible to select a suitable animal model according to the purpose and conditions of an experiment. Several animal studies have shown that G-Rg1 could increase neuron proliferation and survival, alleviating neuronal damage [51, 52]. The apparent neuroprotective effect of G-Rg1 could involve several mechanisms. The accumulation of Aβ, oxidative free radicals, and harmful inflammatory cytokines, as well as excessive apoptosis, could all result in neurological impairment, and G-Rg1 could alleviate these reactions [77]. In addition, G-Rg1 can promote the proliferation of NSCs and attenuate their senescence [30]. Zhu et al. found that G-Rg1 could protect NSCs in the hippocampus of aged rats by reducing the activation of astrocytes and increasing the number of new cells [26]. Some studies have reported that BDNF, a neurotrophin, increased in AD animals after G-Rg1 treatment [49], and this could have a great effect on neuron structure and function [78]. It may also contribute to the effect of reversing long-term potentiation deficits in AD animals [49]. Furthermore, G-Rg1 could increase the expression of synaptic proteins, such as SYN2 and SNP25, in the hippocampus of AD animals [42], which helps to improve neuroplasticity.

To assess different types of memory deterioration during AD, a great number of cognitive behavioral tests could be used. For example, the MWM, Y maze, and radial-arm water maze were mainly created to test spatial memory, object recognition in episodic memory, and fear conditioning in emotional memory. From the results of meta-analysis, we found that G-Rg1 could ameliorate memory deteriorations of different types, including various aspects of learning and memory. Among those memory types, spatial memory is widely assessed in rodent research. However, we must take other forms of memory into account. For instance, episodic and semantic memory are the first types of memory to deteriorate in AD patients and are therefore important to study [79]. Associative memory has been suggested to be paired with working memory, whose deterioration will result in progressive decline of executive function [80].

Nearly all developments in conventional medical treatments cannot be separated from animal research [81]. However, translation from animal tests to the prediction of effects in clinical trials is still a huge challenge [82]. Poor experimental design and a lack of transparent reporting are considered to be the main factors leading to the failure of this translation [83]. The calculation of sample size and the use of blind methods in the induction of the model or the assessment of its outcome are essential for the design of effective studies [84]. In addition, the Animal Research: Reporting of *In Vivo* Experiments (ARRIVE) guidelines could improve the quality of research designs of *in vivo* animal studies, and these have been endorsed by over 300 research journals throughout the world by 2014 [85]. Due to the methodological weaknesses in the included studies, we recommend that future studies on the use of G-Rg1 for cognitive function should refer to these well-established guidelines. Using animals of different sexes can lead to different performances in cognitive behavior [65]. Thus, we suggest choosing a single-sex animal model in future studies. In addition, anesthesia should be selected carefully since some anesthetic compounds such as pentobarbital sodium may result in certain damage to cognitive function [66].

To date, about 40 kinds of ginsenoside compounds have been identified [86]. At least eight ginsenosides; i.e., Rg1, Rb1, Rg2, Rd, Re, Rh1, Rh2, and Rg3, have been reported to improve cognitive function in different animal models. Ginsenosides are generally divided into two groups. The protopanaxatriol group contains Rg1, Rg2, Re, and Rh1, while the protopanaxadiol group contains Rb1, Rd, Rg3, and Rh2 [19]. Individual ginsenosides, which have different chemical structures, have differences in their pharmacology and mechanisms [87]. The most widely studied ginsenoside is Rg1, and this possibly has the greatest protective effects on both memory acquisition and retention in AD animals [25]. The other ginsenosides have been studied much less regarding cognitive impairments than Rg1. These ginsenosides have antioxidant, anti-apoptosis, and anti-inflammatory effects in common, but study of other aspects of their pharmacology has been limited. For example, Rb1 is the second-most comprehensively studied ginsenoside. Pharmacological studies have indicated that Rb1 can increase hippocampal glutamatergic transmission and improve long-term potentiation and synaptic plasticity, suggesting that Rb1 may represent a potential treatment strategy for cognitive impairment [88]. In a few studies, Rd enhanced cognitive performance through estrogen-like activity [89]. Re ameliorates brain insulin resistance and decreases the levels of triglycerides, total cholesterol, and low-density lipoprotein cholesterol, resulting in a protective effect on diabetes-associated cognitive deficits [90, 91].

In future studies, to clarify the different efficacies of different ginsenosides, a new network meta-analysis is needed. In addition, the combined use of two or more kinds of ginsenoside is a novel direction for anti-dementia treatment studies. The composition of isolated ginsenosides is clearer than that of ginseng extract. The use of isolated ginsenosides can help to facilitate an in-depth exploration of related mechanisms. Synergism refers to the combined use of two or more drugs that may have additive pharmacological effects and greater efficacy than individual use of each compound. Shi et al. [40] reported that the synergistic use of G-Rg1 and *Acori graminei* rhizoma attenuates neuron cell apoptosis by promoting the expression of miR-873-5p, and the synergistic use of geniposide and G-Rg1 has been shown to balance microglial TNF-α and transforming growth factor β1 following oxygen–glucose deprivation *in vitro* [92]. Complex pathologic processes are involved in AD, and thus interventions involving multiple targets are necessary.

### Possible mechanisms

**Figure 9:**
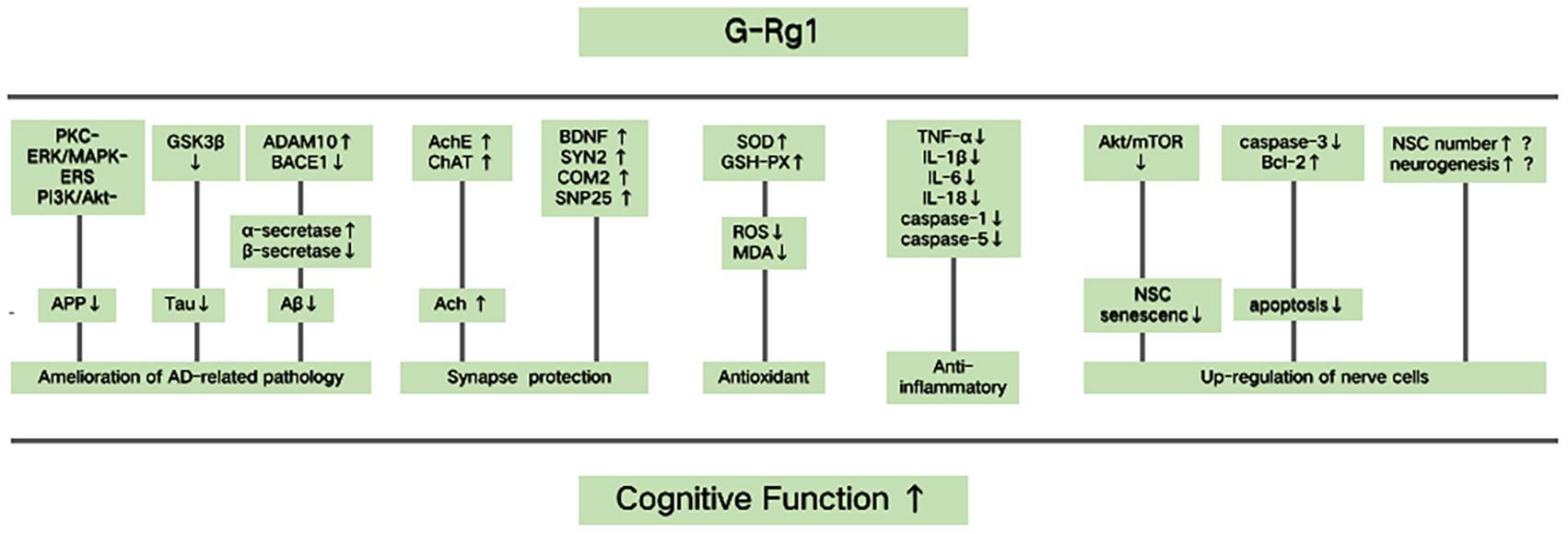
**Possible mechanisms of ginsenoside Rg1 (G-Rg1) in improving cognitive function.** Possible mechanisms for Rg1 improving cognitive function are the following. (1) Rg1 could inhibit the pathogenesis of Alzheimer’s disease (AD). G-Rg1 could promote cleavage of amyloid precursor protein (APP), inhibit the hyperphosphorylation of tau and prevent amyloid - β (Aβ) deposition. This would occur by increasing a disintegrin and metallopeptidase domain 10 (ADAM10) expression and decreasing β-secretase β-site APP-cleaving enzyme 1 (BACE1) expression. (2) Rg1 could offer synapse protection. G-Rg1 could increase the levels of ACh, BDNF, and multiple synaptic proteins, such as synapsin 2 (SYN2), complexin 2 (COM2), and synaptosomal-associated protein 25 (SNP25). (3) Rg1 could increase antioxidant activity. G-Rg1 could increase the activity of SOD and GSH-PX, and could decrease the levels of ROS and MDA. (4) Rg1 could increase anti-inflammatory activity. G-Rg1 could inhibit the expression of TNF-α, decrease the levels of IL-1β, IL-6, and IL-18, and decrease the expression of caspase 1 and caspase 5. (5) Rg1 could up-regulate nerve cells. G-Rg1 treatment delays neural stem cell (NSC) senescence and decreases cell apoptosis, and G-Rg1 treatment increases the number of NSCs and new nerve cells; however, the mechanism for this is not yet clear.

Based on the findings of the included studies, the multitarget mechanisms of G-Rg1 improving cognitive function are as follows. (1) Amelioration of AD-related pathology. Rg1 treatment could increase the expression of ADAM10, an a-secretase that plays a key role in preventing Aβ deposition. In addition, G-Rg1 decreases the expression of BACE1, a b-secretase that exhibits opposing functions to a-secretases [54]. G-Rg1 inhibits the hyperphosphorylation of tau by preventing the activation of the GSK3β pathway [55] and promotes cleavage of APP via its effects on PKC-, ERK/MAPK-, ERS, and PI3K/Akt- dependent signals [53]. (2) Synapse protection. G-Rg1 could inhibit AChE activity and increase choline acetyltransferase activity, maintaining the levels of ACh in cholinergic neurons [31, 47]. G-Rg1 increases the expression of BDNF and multiple synaptic proteins, such as synaptosomal-associated protein 25, synapsin 2, and complexin 2, which help to improve neuron structure and function [42, 49]. (3) Antioxidant activity. G-Rg1 increases the levels of antioxidant enzymes SOD and GSH peroxidase (GSH-PX), inhibits the formation of ROS, and reduces the production of MDA [51, 25]. (4) Anti-inflammatory activities. G-Rg1 could inhibit the expression of TNF-α, decrease the levels of IL-1β, IL-6, and IL-18, and decrease the expression of NLRP1, caspase 1, and caspase 5 in the hippocampus and frontal cortex [25, 37]. (5) Up-regulation of nerve cells. G-Rg1 treatment delays NSC senescence via inhibiting the AKT/mTOR signaling pathway [30]. Through attenuating caspase 3 activity [53] and increasing the expression of Bcl 2, G-Rg1 decreases cell apoptosis. In addition, G-Rg1 treatment could maintain the number of NSCs and increase neurogenesis by increasing the number of new cells [25]; however, the mechanism for this is not yet clear (see Figure 9).

Preclinical systematic review is a common tool in basic life-sciences research, particularly for translating work from the laboratory to human healthcare [93]. The assessment of accumulated animal experiments is helping to rationalize clinical trials, reducing their costs, and reducing the potential risks involved in human tests [94]. Randomized controlled trials have long been regarded as the gold standard when assessing the efficacy and safety of interventions [95]. There have already been several clinical studies assessing the effects of ginseng on cognition [96, 97], and their results have shown that ginseng could improve cognitive function. However, there have been no clinical studies assessing G-Rg1 and cognition. In fact, the effects of G-Rg1 in improving cognitive function have been shown to manifest not only in AD animals, but also in normal animals [98, 99]. Given the obvious neurobehavioral and neurobiochemical effects of G-Rg1, it may prove to have great value in further clinical trials. However, because of the huge gap between animal studies and clinical trials, rigorous RCTs are needed.

## CONCLUSIONS

The present study showed that G-Rg1 could improve learning and memory function in most animal models of AD. The potential mechanisms involved included antioxidant and anti-inflammatory effects, amelioration of AD-related pathology, synapse protection, and up-regulation of nerve cells via multiple signaling pathways.

## MATERIALS AND METHODS

### Search strategy

A total of six English and Chinese electronic databases were searched from their inceptions to January 2019, including PubMed, EMBASE, the Cochrane Library, China National Knowledge Infrastructure, the Wanfang Database, and the VIP Journals Database. The following search terms were used: (Ginseng OR Ginsenoside OR Rg1) AND (memory OR learning OR cognitive OR Alzheimer’s disease OR dementia).

### Eligibility criteria

### Types of studies

Animal studies that assess the effectiveness of G-Rg1 for cognitive function were included, regardless of their language, blinding, or publication status. Case reports, reviews, and protocols were excluded.

### Types of experimental animals

All animal models of Alzheimer’s disease were included, regardless of the animal species, sex, or modeling methods.

### Types of intervention and comparators

The analyzed interventions all included G-Rg1 being received as a monotherapy at any dose. Comparator interventions were isosteric non-functional liquids, such as normal saline or phosphate-buffered saline, or no treatment.

### Types of outcome measures

The primary outcomes were indexes of learning and/or memory-function tests, such as the Morris water maze, Y maze, step-down test, dark-avoidance test, active-avoidance reaction, and fear-conditioning test. The secondary outcome measures were the mechanisms of G-Rg1 for AD.

### Data extraction

The following data were extracted from the included articles by two independent authors: (1) first author’s name and the date of publication; (2) information related to the experimental animals, such as their species, sex, and weight; (3) modeling methods and use of anesthetic; (4) information relating to the treatment group, including therapeutic drug dosage, method of administration, and duration of treatment, with the same information being recorded for the control group; and (5) outcomes and intergroup differences for each outcome measure. The data relating to the highest dose were included when the treatment groups included various doses of the drug. The result of the last time point was included when the data were expressed for different times. When published outcome data were displayed graphically, we attempted to contact the author for specific information. Digital ruler software was applied when failing to receive a response. Where several articles were published from a single study, we chose the article with the largest sample or the earliest publication.

### Quality assessment

Two authors independently assessed the methodological quality of the included articles, referring to the Collaborative Approach to Meta-Analysis and Review of Animal Data from Experimental Studies [100]. One point was given for each criterion based on written evidence. Each study was then given an aggregate quality score after completing the evaluation of ten criteria. The divergences in the process were finally settled after discussion among the authors of the present study or by consultation with the corresponding authors.

### Statistical analysis

A meta-analysis was carried out using the RevMan 5.3 software package. The outcome of each indicator was considered as continuous data, and a fixed effects model and the SMD were used to estimate the combined overall effect sizes. The efficacy of G-Rg1 in improving learning and memory function was assessed utilizing the SMD with a 95% confidence interval. Funnel plots were used to evaluate publication bias. To clarify the effect that mixed factors played on the outcome measure, sensitivity analysis and subgroup analysis were performed according to several variables, including animal species and sex and modeling methods. We used the I^2^ statistic to assess the heterogeneity among individual studies. Probability values *P* < 0.05 were considered significant.

## Supplementary Material

Supplementary Table 1

Supplementary Table 2
