# Supplementary material for: Preclinical systematic review of ginsenoside Rg1 for cognitive impairment in Alzheimer’s disease
Source: Aging (Albany NY). 2021 Mar 3;13(5):7549–69. doi: 10.18632/aging.202619 (PMC7993717; doi:10.18632/aging.202619)
Supplement: Supplementary Table 1 [file aging-13-202619-s001.docx]

**Table 1. Characteristics of the included studies.**

| Study (Name, Years) | Animal (Species, Sex, Weight, N) | Anesthetic | Model (Method) | Experimental Group | Control Group | Outcome Measure | Intergroup Differences |
| --- | --- | --- | --- | --- | --- | --- | --- |
| Chen et al. 2005 | SD Rats Male/Female 180-220  9*10 | - | Scopolamine | 20mg/kg  ig  14d | Saline | 1.Y-Maze (A: Learn, B: Memory) 2.AChE | 1.A:P<0.01, B:P<0.01 2.P<0.01 |
| Chen et al. 2011 | SD Rats  Female 180-220  4*10 | Pentobarbital Sodium | Hippocampus Electrical Injury | 50mg/kg  ig 14d | Saline | 1.Morris Maze 2.Number of Neurons (Using optical microscope) | 1.P<0.05 2.P<0.05 |
| Chen et al. 2017 | C57BL/6J Mice  ? ?  3*10 | - | D-Gal | 20mg/kg  ip 28d | Saline | 1. Morris Maze 2.NSCs 3.MDA, SOD, GSH-Px 4.ROS | 1.P<0.05 2.P<0.05 3.P<0.05 4.P<0.05 |
| Fang et al. 2012 | Tg mApp Mice  ? ?  4*（5-7） | - | Transgenic | 10mg/kg  ip 3m | PBS | 1.Radial Arm Water Maze 2.Aβ 3.γ Secretase  4.AChE  5.CREB | 1.P<0.05 2.P<0.01 3.P<0.05 4.P<0.01 5.P<0.05 |
| Hu et al. 2004 | Wistar Rats  Male 180-220  4*10 | - | Chronic Stress | 10,50mg/kg  ip 7d | No Treatment | 1.Morris Maze 2.Number of Neurons (using optical microscope) 3.Ca^2+^ | 1.P<0.05 2.P<0.05 3.P<0.01 |
| Li et al. 2007 | Wistar Rats  ? 450  5*10 | Chloral Hydrate | Quinolinic Acid | 20,40mg/kg  ig 16d | Distilled Water | 1.Step-down Test 2.Water Maze | 1.P<0.001 2.P<0.01 |
| Li et al. 2014 | SD Rats  Male 150-210  4*10 | - | D-Gal | 20mg/kg  ig 27d | Saline | 1.Morris Maze 2.SA-β-gal 3.IL-1, IL-6 4.SOD, GSH 5.Telomere | 1.P<0.05 2.P<0.05 3.P<0.05 4.P<0.05 5.P<0.05 |
| Li et al. 2015 | Kunming Mice  Male 18-22  9*10 | Chloral Hydrate | AΒ1-42 | 7.5,15,30mg/kg ip 1m | Saline | 1.Morris Maze 2.Ab1–42 3.Metabolite Profiles | 1.P<0.01 2.- 3. P<0.05 |
| Li et al. 2016a | APP/PS1 Mice  ? ?  4*20 | - | Transgenic | 0.1,1,10mg/kg  ip 1m | Saline | 1.Fear Conditioning Experiment 2.LTP 3.APP, CTFs, P‑Tau 4.Aβ1-42  5.BDNF | 1.P<0.05 2.P>0.05 3.P<0.05 4.P<0.01 5.P<0.05 |
| Li et al. 2016b | APP/PS1 Mice Male 18-22  4*10 | - | Transgenic | 30 mg/kg  ip 1m | Saline | 1.Morris Maze 2.Aβ 3.Plasma Metabolites | 1.P<0.01 2.- 3.P<0.05 |
| Liu et al. 2015 | Nestin-GFP Mice  ?  ?  5*6 | - | D-Gal | 60mg/kg  ip 30d | Saline | 1.Morris Maze 2.PCNA+.DCX+.Tubulin+.GFAP+ 3.SOD, MDA, BDNF, GDNF | 1.P<0.05 2.- 3.P<0.05,P<0.01,P<0.05,P<0.05 |
| Nie et al. 2017 | APP/PS1/Tau Mice Female ?  3*(10–15) | - | Transgenic | 20mg/kg  ip 6w | Saline | 1.Anti-Anxiety：(A:Open Field Test, B:Elevated Plus Maze Test) 2.Tail Suspension Test 3.Morris Water Maze 4. SNP25, SYN2, Complexin 2 | 1.A:P<0.05, B:P>0.05 2.P<0.05 3.P<0.05 4.P<0.05 |
| Peng et al. 2011 | SD Rats  Male 212-253  3*5 | - | D-Gal | 20mg/kg  ip 28d | Saline | 1.Morris Maze 2.SOD, MDA 3.expression of P16, P21 | 1.P<0.05 2.P<0.05 3.P<0.05 |
| Quan et al. 2013 | SD Rats  Male 210-230  4*10 | Chloral Hydrate | AΒ1-42 | 10mg/kg  ip 28D | Saline | 1.Morris Maze 2.Aβ, PPARγ, IDE | 1.P<0.05 2.P<0.05 |
| Shi et al. 2008 | SAMP8 Mice  Male 29-41  10*(6-8) | - | Transgenic | 2.5,5,10mg/kg  ip 3m | Saline | 1.Y-Maze (A: Learn, B: Memory) 2.Step-down Test 3.Aβ 4.CREB 5.BDNF 6.PKA IIA | 1.A,B:P<0.01 2.P<0.01 3.P<0.05 4.P<0.05 5.P<0.05 6.P<0.05 |
| Shi et al. 2012 | Wistar Rats  Female ?  4*10 | ？ | Ovariectomy | 10mg/kg  iv 8w | Saline | 1. Morris Maze 2.sAPPα   3.Aβ1-42 | 1.P<0.01 2.P<0.01  3.P<0.01 |
| Shi et al. 2018 | SAMP8 Mice  ？ ？  5*10 | - | Transgenic | 7.5 mg/kg  ig 3w | Saline | 1.Morris Maze 2.Expression of Mir‑873‑5p and HMOX1 | 1.P>0.05 2.P>0.05 |
| Song et al. 2013 | SD Rats  Male 200-240  7*12 | Chloral Hydrate | Okamoto Acid | 5,10,20mg/kg  ig 25d | Distilled Water | 1.Morris Maze 2.Tau 3.Gsk3Β | 1.P<0.05 2.P<0.001 3.P<0.001 |
| Wang et al. 2001 | KM Mice  Male 32-36  4*(10-11) | Ether | AΒ25-35 | 5,10mg/kg  ip 10d | Saline | 1.Morris Maze 2.Dark Avoidance Test 3.ChAT, AChE | 1.P<0.01 2.P<0.05 3.P<0.01, P<0.05 |
| Wang et al. 2010 | ICR Mice  Male 20–22  12*10 | - | Scopolamine | 6,12mg/kg  ip 7d | Saline | 1.Locomotor Activity Test 2.Step-Down Test 3.Morris Maze 4.AchE 5.Ach and 5-HT | 1.P>0.05 2.P<0.05 3.P<0.01 4.P<0.05 5.P<0.05 |
| Wang et al. 2014b | KM Mice  Male 25-30  4*15 | - | Chronic Stress | 2,5mg/kg  ig 8w | Distilled Water | 1.Morris Maze 2.Neuronal Degeneration 3.ROS 4.SOD, 8-ohdg  5.P47phox 6.Expression of RAC1 7.P47phox, RAC1 And NOX2 | 1.P>0.05 2.- 3.P<0.05 4.P<0.05 5.P<0.05 6.P<0.05 7.P<0.05 |
| Wu et al. 2007 | Wistar Rats  Male 250-300  3*10 | Chloral Hydrate | Hippocampus Injury | 5mg/kg  ip 30d | Saline | 1.Morris Maze 2.TPKA | 1.P<0.05 2.P<0.05 |
| Wu et al. 2011 | SD Rats  Male 250-400  5*15 | Chloral Hydrate | Hippocampus Injury | 5mg/kg  ip 4w | Saline | 1.Morris Maze 2.NGF mRNA | 1.P<0.05 2.P<0.05 |
| Xiang et al. 2017 | Nestin-GFP Mice  ? 19-27  4*10 | - | D-Gal | 40 mg/kg  ip 26d | Saline | 1. Morris Maze 2.Nestin-GFP (DG area)   3.SA-β-gal (CA3 Area) 4.SOD, T-AOC 5.IL-1Β, IL-6, TNF-Α  6.P53,P21 Protein | 1.P<0.05 2.P<0.05 3.P<0.05 4.P<0.05 5.P<0.05 6.P<0.05 |
| Yang et al. 2013 | C57BL/6J Mice Female ?  2*(12-16) | - | Aged | 6mg/kg/3d Ig 12m | Saline | 1.Life 2.Y Maze 3.Morris Maze 4.H_2_0_2_, SOD 5.ChAT 6.mTOR | 1.P<0.05 2.P<0.01 3.P<0.05 4.P<0.01, P<0.05 5.- 6.P<0.01 |
| Ye et al. 2017 | SD Rats  Male 180-220  3*12 | ? | Aβ1-42 | 50mg/kg  ig 30d | Saline | 1.Morris Maze 2.Aβ  3.Cell Apoptosis (TUNEL) | 1.P<0.01 2.- 3.P<0.01 |
| Yuan et al. 2016 | APP/PS1 Mice  ？ 20-25  4*6 | - | Transgenic | 100 mg/kg  ip 8w | Saline | 1.Morris Maze 2.Serum T-SO, GSH  3.Hippocampus T-SOD, GSH, GSH-Px, MDA | 1.- 2.P<0.05 P>0.05 3.P>0.05 P>0.05 P<0.05 P<0.05 |
| Zhang et al. 2012 | Wistar Rats  Female 260-300  8*12 | Chloral Hydrate | Ovariectomy+D-Gal | 5,10,20mg/kg  ip 6w | Saline | 1.Morris Maze 2.Aβ1-42  3.ADAM 10 4.BACE1 5.Caspase 3 | 1.P<0.05 2.P<0.05 3.P<0.05 4.P<0.05 5.P<0.05 |
| Zhang et al. 2017a | SAMP8 Mice  Male ?  3*15 | - | Transgenic | 15 mg/kg/D  Oral Gavage 4m | Distilled Water | Morris Maze | P<0.05 |
| Zhang et al. 2017b | ICR Mice  Male  25-30  6*10 | - | Dexamethasone | 1,2,4mg/kg  ig  28d | Distilled Water | 1. Open field test 2. Neuronal injury (HE staining) 3. MAP2 4. GR 5. NLRP-1 and ASC 6. caspase-1 and caspase-5 7. IL-1β, IL-18 | 1. P<0.01 2. P<0.01 3. - 4. P<0.01 5. P<0.01 6. P<0.05 7. P<0.05 8. P<0.05 |
| Zhou et al. 2011 | C57BL/6J Mice Female 18-22  4*10 | Chloral Hydrate | AΒ25-35 | 10 mg/ kg  ip 14d | Saline | 1.Morris Maze 2.Bcl-2 | 1.P<0.05 2.P<0.05 |
| Zhu et al. 2014 | SD Rats  Male ?  4*15 | - | D-Gal | 20 mg/kg  ip 28d | Saline | 1.Morris Maze 2.SA-B-Gal 3.Telomere Lengths 4. Brdu (in Dentate Gyrus) 5.Expression of SOX2, Nestin And Aeg1 6.NSCs 7.SOD, MDA, GSH, GSH-Px 8.IL-1b, IL-6 ,TNF-α | 1.P<0.05 2.P<0.01 3.P<0.05 4.P<0.05 5.P<0.05 6.P<0.05 7.P<0.05 8.P<0.05 |

Note: Aβ, the amyloid β; Ach, acetylcholine; AchE, Acetylcholinester ase; ADAM 10, a disintegrin and metallopeptidase domain 10; APP, amyloid precursor protein; BACE 1, β-site APP-cleaving enzyme 1; BDNF, brain derived neurotrophic factor; CREB, cAMP - response element binding protein; EL, escape latency; GSH, glutathione; GSH-Px, glutathione peroxidase; IDE, insulin-degrading enzyme; Ig, intragastrical administration; Ip, intraperitoneal administration; KM mice, KunMing mice; LTP, long-termpotentiation; MDA, malondialdehyde; NSCs: neural stem cells PBS,Phosphate Buffered Saline; ROS, reactive oxygen species; SD rats, Sprague Dawley rats; SNP, synaptosomal-associated protein; SOD, superoxide dismutase; SYN, synapsin; TNF-α, Tumor Necrosis Factor α.
