# Supplementary material for: Preclinical systematic review of ginsenoside Rg1 for cognitive impairment in Alzheimer’s disease
Source: Aging (Albany NY). 2021 Mar 3;13(5):7549–69. doi: 10.18632/aging.202619 (PMC7993717; doi:10.18632/aging.202619)
Supplement: Supplementary Table 2 [file aging-13-202619-s002.docx]

**Table 2: Statement of the Quality control of G-Rg1**

| **Study** | **Source** | Species, **Concentration** | Quality control reported? (Y/N) | Chemical analysis reported? (Y/N) |
| --- | --- | --- | --- | --- |
| Chen et al. 2005 | Jilin Hongjiu Biological Technology Co., Ltd (Jilin, China) | Panax, Ginseng C. A. Mey. Intragastric Injection at 20mg/kg | Y – According to Lot number (20040504) | Y– HPLC/UV |
| Chen et al. 2011 | Shanghai Tongtian Biological Technology Co., Ltd (Shanghai, China) | Panax, Ginseng C. A. Mey. Intragastric Injection at 50mg/kg | Y – According to Lot number (07080421) | Y – HPLC |
| Chen et al. 2017 | Jilin Hongjiu Biological Technology Co., Ltd (Jilin, China) | Panax, Ginseng C. A. Mey. Intraperitoneal Injected at 20mg/kg | Y – According to Lot number (RSZD-121106) | Y – HPLC/UV |
| Fang et al. 2012 | The Department of Biochemistry of Jilin University, China. | Panax, Ginseng C. A. Mey. Intraperitoneal Injected at 10mg/kg | N | N |
| Hu et al. 2004 | Not Reported | Panax, Ginseng C. A. Mey. Intraperitoneal Injected at 10,50mg/kg | N | N |
| Li et al. 2007 | The Chinese National Institute for The Control of Pharmaceutical And Biological Products (Beijing, China) | Panax, Ginseng C. A. Mey. Intragastric Injection at 20, 40mg/kg | N | Y – HPLC |
| Li et al. 2004 | Jilin Hongjiu Biological Technology Co., Ltd (Jilin, China) | Panax, Ginseng C. A. Mey. Intragastric Injection at 20mg/kg | N | Y – HPLC/UV |
| Li et al. 2015 | Shanghai Yuanye Bio-Technology Co., Ltd (Shanghai, China) | Panax, Ginseng C. A. Mey. Intraperitoneal Injected at 7.5,15,30mg/kg | N | N |
| Li et al. 2016a | Sigma‑Aldrich, St. Louis, MO, USA | Panax, Ginseng C. A. Mey. Intraperitoneal Injected at 0.1, 1, 10mg/kg | N | Y – HPLC |
| Li et al. 2016b | Shanghai Yuanye Bio-Technology Co., Ltd (Shanghai, China). | Panax, Ginseng C. A. Mey. Intraperitoneal Injected at 30 mg/kg | N | N |
| Liu et al. 2015 | Xi’an Haoxuan Biological Technology Co., Ltd (Xi’an, China) | Panax, Ginseng C. A. Mey. Intraperitoneal Injected at 60mg/kg | N | Y – UV |
| Nie et al. 2017 | Prof. Zhendan He, College of Life Sciences, Shenzhen University | Panax, Ginseng C. A. Mey. Intraperitoneal Injected at 20mg/kg | N | N |
| Peng et al. 2011 | Jilin Hongjiu Biological Technology Co., Ltd (Jilin, China) | Panax, Ginseng C. A. Mey. Intraperitoneal Injected at 20mg/kg | N | Y – HPLC/UV |
| Quan et al. 2013 | Hongjiu Biotech. Co., Ltd. (Jilin, China) | Panax, Ginseng C. A. Mey. Intraperitoneal Injected at 10mg/kg | N | Y – HPLC |
| Shi et al. 2008 | Organic Chemistry Laboratory of Norman Bethune Health Science Center of Jilin University | Panax, Ginseng C. A. Mey. Intraperitoneal Injected at 2.5, 5, 10mg/kg | N | N |
| Shi et al. 2012 | Sigma (St. Louis, MO, USA) | Panax, Ginseng C. A. Mey. Intravenous Injection at 10mg/kg | N | Y - HPLC |
| Shi et al. 2018 | ESITE Biotech Co. Ltd., (Chengdu, China) | Panax, Ginseng C. A. Mey. Intragastric Injection at 7.5 mg/kg | N | N |
| Song et al. 2013 | Jilin University (Jilin, China) | Panax, Ginseng C. A. Mey. Intragastric Injection at 5, 10, 20mg/kg | N | N |
| Wang et al. 200  1 | Organic Chemistry Laboratory of Norman Bethune Health Science Center of Jilin University | Panax, Ginseng C. A. Mey. Intraperitoneal Injected at 5, 10mg/kg | N | N |
| Wang et al. 2010 | Shanghai Innovative Research Center of Traditional Chinese Medicine (Shanghai, China) | Panax, Ginseng C. A. Mey. Intraperitoneal Injected at 6, 12mg/kg | N | Y - HPLC |
| Wang et al. 2014b | The Institute of Pharmaceutical Research of Xiehe Medical University of China | Panax, Ginseng C. A. Mey. Intragastric Injection at 2, 5mg/kg | N | Y – HPLC |
| Wu et al. 2007 | Organic Chemistry Laboratory of Norman Bethune Health Science Center of Jilin University | Panax, Ginseng C. A. Mey. Intraperitoneal Injected at 5mg/kg | N | N |
| Wu et al. 2011 | Not Reported | Panax, Ginseng C. A. Mey. Intraperitoneal Injected at 5mg/kg | N | N |
| Xiang et al. 2017 | Jilin Hongjiu Biological Technology Co., Ltd (Jilin, China) | Panax, Ginseng C. A. Mey. Intraperitoneal Injected at 40 mg/kg | N | Y – HPLC/UV |
| Yang et al. 2013 | Organic Chemistry Laboratory, College of Basic Medical Sciences of Jilin University (Jilin, China) | Panax, Ginseng C. A. Mey. Intragastric Injection at 6mg/kg/3D | N | N |
| Ye et al. 2017 | Shanghai Tongtian Biological Technology Co., Ltd (Shanghai, China) | Panax, Ginseng C. A. Mey. Intragastric Injection at 50mg/kg | Y – According to Lot number (12080421) | Y - HPLC |
| Yuan et al. 2016 | Xi’an Haoxuan Biological Technology Co., Ltd (Xi’an, China) | Panax, Ginseng C. A. Mey. Intraperitoneal Injected at 100 mg/kg | N | Y - UV |
| Zhang et al. 2012 | The National Institute for The Control of Pharmaceutical And Biological Products (Beijing, China) | Panax, Ginseng C. A. Mey. Intraperitoneal Injected at 5, 10, 20mg/kg | N | Y – HPLC |
| Zhang et al. 2017a | The Chinese National Institute for The Control of Pharmaceutical And Biological Products (Beijing, China) | Panax, Ginseng C. A. Mey. Oral Gavage at 15 mg/kg/D | N | Y – HPLC |
| Zhang et al. 2017b | The Institute of Pharmaceutical Research of Xiehe Medical University of China | Panax, Ginseng C. A. Mey. Intragastric Injection at 2, 5mg/kg | N | Y – HPLC |
| Zhou et al. 2011 | Norman Bethune Health Science Center of Jilin University | Panax, Ginseng C. A. Mey. Intraperitoneal Injected at 10 mg/ kg | N | N |
| Zhu et al. 2014 | Xi’an Haoxuan Biological Technology Co., Ltd (Xi’an, China) | Panax, Ginseng C. A. Mey. Intraperitoneal Injected at 20 mg/kg | Y – According to Lot number (RSZD-121106) | Y - UV |
